# Cai’s herbal tea enhances mitochondrial autophagy of type 1 diabetic mellitus β cells through the AMPK/mTOR pathway and alleviates inflammatory response

**DOI:** 10.1007/s00592-024-02316-y

**Published:** 2024-07-02

**Authors:** Hongchun Li, Yanfei Gao, Mengdi Li, Yue Dong, Jie Chen, Bingyue Zhang, Kaiqiang Li, Yuqun Cai

**Affiliations:** 1 Diabetes and Obesity Clinic, Tongde Hospital of Zhejiang Province, Xihu District, Hangzhou, 310012 Zhejiang China; 2 Rehabilitation Medicine Center, Tongde Hospital of Zhejiang Province, Xihu District, Hangzhou, 310012 Zhejiang China; 3 Department of Integrated Traditional Chinese and Western Medicine, Zhejiang Provincial People’s Hospital, Gongshu District, Hangzhou, 310014 China; 4 Laboratory Medicine Center, Allergy Center, Department of Transfusion Medicine, Zhejiang Provincial People’s Hospital (Affiliated People’s Hospital), Gongshu District, Hangzhou, 310014 China

**Keywords:** Animal study, Cai’s herbal tea, Type 1 diabetes mellitus, AMPK-mTOR pathway, Autophagy

## Abstract

**Background:**

This study investigates the therapeutic mechanisms of Cai’s Herbal Tea in Type 1 Diabetes Mellitus (T1DM) mice, focusing on its effects on mitochondrial change and autophagy via the AMP-activated protein kinase (AMPK)—mammalian target of rapamycin (mTOR) pathway.

**Methods:**

The composition of Cai’s Herbal Tea was analyzed by Ultra-High Performance Liquid Chromatography-Quadrupole Time of Flight Mass Spectrometry (UHPLC-Q/TOF-MS). C57BL/6 mice and Min6 pancreatic beta cells were divided into control, diabetic mellitus (DM)/high glucose (HG), and treatment groups (low, medium, and high doses of Cai’s Tea, and Metformin). Key physiological parameters, pancreatic islet health, Min6 cell morphology, viability, and insulin (INS) secretion were assessed. Small Interfering RNA-AMPK (si-AMPK) was utilized to confirm the pathway involvement.

**Results:**

Cai’s Herbal Tea improved body weight, pancreatic islet pathological injury, and INS secretion whereas reduced total triglycerides, fasting blood sugar, and Interferon gamma (INF-γ) in T1DM mice, particularly at higher doses. In Min6 cells, Cai’s Tea mitigated HG-induced damage and proinflammatory response, enhancing cell viability and INS secretion. Notably, it reduced swelling and improved cristae structure in treated groups of mitochondria and promoted autophagy via the AMPK-mTOR pathway, evidenced by increased LC3II/LC3I and P-AMPK/AMPK ratios, and decreased P-mTOR/mTOR and P62 expressions in pancreatic islet β-cells. Furthermore, these effects were converted by si-AMPK interference.

**Conclusion:**

Cai’s Herbal Tea exhibits significant therapeutic efficacy in T1DM mice by improving mitochondrial health and inducing autophagy through the AMPK-mTOR pathway in pancreatic islet β-cells. These findings highlight its potential as a therapeutic approach for T1DM management.

**Supplementary Information:**

The online version contains supplementary material available at 10.1007/s00592-024-02316-y.

## Background

Type 1 diabetes mellitus (T1DM) is an acute chronic disease characterized by T lymphocyte-mediated autoimmune response against pancreatic islet β-cells, leading to islet damage and absolute insulin (INS) deficiency [1–3]. Inflammatory stress plays a crucial role in its pathogenesis [4, 5]. Inflammatory damage is often associated with β-cell dysfunction [6]. Studies indicate that inflammation plays a role in the pathogenesis of certain glucose disorders in adults. Pancreatic β-cells exposed to cytokines, such as interleukin-1β (IL-1β), are thought to contribute to β-cell apoptosis [7]. Although the exact molecular mechanisms are unclear, excessive production of free radicals may lead to β-cell inflammatory damage [8]. Inflammation negatively impacts mitochondria in β-cells, leading to disturbances in energy metabolism, reduced glucose-stimulated INS secretion, and activation of apoptosis [9]. Therefore, inhibiting inflammation and protecting mitochondrial function in β-cells are potential therapeutic approaches for diabetes.

Autophagy is an endogenous mechanism that reduces lipid peroxidation and promotes β-cell survival [10, 11]. It is a specialized form of ‘eating’ degradation occurring in secretory cells, where secretory granules directly fuse with lysosomes [12]. Although the mechanisms of autophagy are not fully understood, this process plays a key role in the regulation of INS granules [13, 14]. Mitophagy, a subtype of autophagy, selectively removes damaged mitochondria and acts as a protective mechanism to reduce oxidative stress and inflammatory responses in cells. Dysregulation of mitophagy is linked to the pathogenesis of various metabolic and age-related diseases, including diabetes. In mice, a high-fat diet leads to reduced mitophagy, inflammatory responses, and ultimately impaired cellular secretion [15]. The T1DM susceptibility gene CLEC16A, encoding an E3 ubiquitin ligase, controls mitochondrial autophagy flux in β-cells [16], indicating a critical role of mitophagy in maintaining β-cell function. In fact, diabetes-associated intronic polymorphisms in the CLEC16A gene region lead to reduced expression of CLEC16A mRNA in human islet cells, resulting in β-cell dysfunction and poor glycemic control [16]. Thus, mitophagy promotes β-cell survival and prevents diabetes by counteracting inflammatory damage. Targeting this pathway may prevent β-cell exhaustion in diabetes.

AMP-activated protein kinase (AMPK) is a nutrient-sensitive kinase activated by phosphorylation during metabolic processes like fatty acid oxidation, glucose uptake, and glycolysis under low energy states. AMPK has been shown to actively induce autophagy, and reduced AMPK is associated with the pathogenesis of diabetes [17]. Importantly, AMPK promotes autophagy under glucose starvation by directly activating Unc-1 (autophagy activating kinase 317 (ULK777) through phosphorylation at Ser51 and Ser1. Conversely, under nutrient-rich conditions, high mTORC1 activity prevents ULK1 activation by phosphorylating ULK1 at Ser1 and disrupting the interaction between ULK757 and AMPK, thus reducing AMPK-ULK1 signal-mediated autophagy induction [18]. Additionally, activation of AMPK can phosphorylate tuberous sclerosis complex 2 (TSC2) and activated TSC2 can inhibit mTORC1-induced autophagy [19]. A recent study reported that AMPK also contributes to autophagosome maturation and lysosomal fusion in HEK293 cells [20]. Therefore, AMPK is a key regulator of autophagy, and AMPK-induced autophagy is a protective mechanism in diabetes.

The Cai’s herbal tea formula (comprising Astragalus (*Astragalus membranaceus)*, Atractylodes (*Atractylodes macrocephala*), Ophiopogon (*Ophiopogon japonicus*), Cornus (*Cornus officinalis*), and Mulberry leaf (*Morus alba*)) can effectively reduce blood glucose levels in patients when combined with a health management plan [21]. Astragalus, a commonly used herb, is sweet and warm in nature and is known for its functions of invigorating Qi, consolidating the exterior, supplementing Qi to raise Yang, and supporting the primary Qi [21]. It has been reported to have a bidirectional regulatory effect on blood sugar and significantly reducing blood sugar levels after glucose loading and counteracting adrenaline-induced hyperglycemia and phenformin-induced hypoglycemia in mice [22]; it can improve material metabolism and chronic complications of diabetes [23]. Some studies confirm that Astragalus polysaccharides can regulate blood sugar and prevent T1DM [23] and inhibit inflammatory responses in diabetes [24, 25], and also alleviate glucose toxicity in cultured mouse cells by the activation of AMPK [26]. However, whether Cai’s herbal tea formula can inhibit the progression of diabetes and the mechanisms involved, including molecular pathways, still require in-depth research. Therefore, this study hypothesizes that Cai’s herbal tea formula can enhance mitochondrial autophagy in β-cells of T1DM through the AMPK/mTOR pathway, alleviate inflammatory damage, and explore its connection with T1DM targets through pharmacological spectrum analysis, systematically examining the effects of Cai’s herbal tea on T1DM at both the whole animal and in vitro cellular levels, with the aim of providing a theoretical basis for further clinical prevention and treatment.

## Methods

### Ultra performance liquid chromatography-quadrupole/time of flight-mass spectrometry (UPLC-Q/TOF-MS) analysis

Component analysis of Cai’s herbal tea was conducted by UPLC-Q/TOF-MS involved the SCIEX Quadrupole Time of Flight 6600 Mass Spectrometer and Shimadzu Nexera X2 Liquid Chromatography-30 Analytical Detector UHPLC System, among other equipment. Reagents included methanol, acetonitrile, formic acid, and Milli-Q water. Chromatographic conditions use an ACQUITY Ultra Performance Liquid Chromatography High Strength Silica T3 column with a mobile phase of formic acid in water and acetonitrile, following a specific gradient elution program. Mass spectrometry employs a TurboIonSpray ion source with both positive and negative ion scanning modes, and specific gas, temperature, and voltage settings. The Time-of-FlightMass Spectrometry (TOF MS) scans a range of 50–1500 Da, with Information Dependent Acquisition (IDA) in high sensitivity mode for secondary Tandem MS/MS. Data analysis was conducted using SCIEX OS software, utilizing mass accuracy, retention time, isotope, and compound library matching, focusing on the TCM MS/MS Library with over 1000 TCM compounds for substance screening.

### Experimental animals

Thirty-six male C57BL/6 mice, each weighing 22–24 g, 8 weeks old, were purchased from Shanghai SLAC Laboratory Animal Co., Ltd., under the animal production license number SCXK (Hu) 2017-0005. During the breeding period, the animals were kept during behavioral adaptive training fed a standard rodent pellet diet, and provided with ad libitum water access, under a 12-h light/dark cycle at Zhejiang Eyong Pharmaceutical Research and Development Co., Ltd. and the license number is SYXK (Zhe) 2021-0003. All animal studies met the guidelines of the China National Council on Experimental Animal Care and Use. Study procedures were approved by the Ethics Committee of Zhejiang Eyong Pharmaceutical Research and Development Center (SYXK (Zhe) 2021-0033).

### T1DM mouse model

Streptozotocin (STZ) (S0130-500MG, sigma) 50 mg/kg intraperitoneal injection to mice once daily for 5 consecutive days according to the previous study[27, 28]. Blood glucose levels were monitored every 3 days using a glucometer for 2 weeks post-STZ administration. Mice with blood glucose > 320 mg/dl were classified as T1DM [27].

### Drug preparation

Cai’s herbal tea contains Astragalus (*Astragalus membranaceus)* 2 g, Atractylodes (*Atractylodes macrocephala*) 2 g, Ophiopogon (*Ophiopogon japonicus*) 2 g, Cornus (*Cornus officinalis*) 2 g, and Mulberry leaf (*Morus alba*) 6 g. Take 500 g of ground powder medicine, soak in 4 L distilled water, boil for 2 h, filter the extract, and then boil again with 4 L distilled water and filter. Combine the two extracts, and concentrate to obtain Cai’s herbal tea extract with a concentration of 1 g/ml for subsequent experiments.

### Drug Intervention

Cai’s herbal tea calculation (adult dose 40 g/70 kg/d [20] converted to mouse dosage based on body surface area, approximately 9.1 times the human dose, i.e., 5.2 g/kg/d). Mice were administered orally once daily for 12 weeks at different concentrations: 5.2 g/kg/d for low dose (cai-L group), 10.4 g/kg/d for medium dose (cai-M group), and 20.8 g/kg/d for high dose (cai-H group). Metformin (200 mg/kg/d, orally, once daily for 12 weeks) as a positive control [29]. Namely, control, DM, DM + cai-L (5.2 g/kg/d), DM + cai-M (10.4 g/kg/d), DM + cai-H (20.8 g/kg/d), DM + Metformin (200 mg/kg/d) groups.

### Sample collection

After the last dose, mouse weight, and fasting blood glucose were measured after overnight fasting, followed by the collection of blood samples and centrifugation to obtain serum for the measurement of total triglycerides (TG) and total cholesterol (TC) levels. Mice were then anesthetized with isoflurane for pancreatic islet collection, some for slicing, and some for molecular experiments.

### Enzyme-linked immunosorbent assay (ELISA)

The Mouse Interferon Gamma (IFN-γ, MM-0182M2), Mouse Interleukin 4 (IL-4, MM-0165M2), and Mouse INS (RX202485M) assays were prepared. Mouse serum samples and the provided standards were added to the appropriate wells for 2-h incubation at room temperature. After incubation with samples and standards, the detection antibody that is specific to IFN-γ, IL-4, or INS was added as required. After the incubation with the detection antibody, the substrate solution was introduced to each well for incubation until the desired color developed. Finally, the stop solution was added to each well to halt the enzymatic reaction, and the absorbance in each well was accordingly recorded using an ELISA plate reader at the wavelength specified in the kit instructions.

### Hematoxylin and eosin (HE) staining

The tissue sections were serially dyed with Hematoxylin (Sigma, H3136) for 5–10 min and rinsed in running tap water to remove excess stain and to blue the nuclei. Subsequently, the slides were immersed in the differentiation solution (Shanghai Ruiyu Biotech, Bry-0001-03) to remove excess Hematoxylin and rinsed again in tap water. Afterward, the tissue sections were stained with Eosin (Sigma, E4009) for 1–5 min, dehydrated through graded alcohols (70%, 95%, 100%), and transparentized in xylene. For the visualization, the slides were mounted with a suitable mounting medium and the stained parts were examined under the Nikon Eclipse Ci-L optical microscope. The images were finally captured using the Nikon DS-Fi2 imaging system for detailed analysis of the pancreatic islet pathology.

### Immunohistochemistry (IHC) staining

This part started with the deparaffinized, rehydrated tissue sections. Firstly, the antigen retrieval was carried out using a Tris-EDTA buffer (Sigma). Secondly, the sections were heated in the buffer, typically in a microwave or pressure cooker, to unmask the antigen sites. The sections were then treated with 3% H_2_O_2_ (National Medicines Corporation) to block endogenous peroxidase activity. This step prevents non-specific staining. Non-specific binding sites were blocked with BSA (Sigma, B2064), which hence reduces background staining. The sections were incubated with the INS Antibody (Affinity, AF5109) diluted 1:100 overnight at 4 °C. After washing, the sections were additionally incubated with the Goat Anti-Rabbit IgG H&L (HRP) pre-adsorbed secondary antibody (Abcam, ab97080) diluted at 1:5000. A chromogen substrate (DAB) was applied for HRP. The sections were counterstained with a mild nuclear stain (Hematoxylin) to provide a contrasting background, dehydrated through graded alcohols, transparentized in xylene, and mounted with the mounting medium in the end.

### Transmission electron microscopy (TEM)

1 mm^3^ tissue samples or approximately 10^7^ cells were routinely collected and fixed immediately in 2.5% glutaraldehyde for 24 h, followed by the removal of the fixative, the washing in PBS buffer for 6 h, and the fixation in 1% osmium tetroxide for 2 h. Then, Min6 cells collected were fixed in (a) 2.5% glutaraldehyde for 4 h, (b) 30% and 50% ethanol for 10 min, (c) 70% ethanol with uranyl acetate (pre-embedding staining) for 3 h or overnight, (d) 80% and 95% ethanol for 10 min and (e) 100% ethanol twice (50 min for each). The samples were with propylene oxide for 30 min and incubated propylene oxide and epoxy resin 1:1 for 2 h. Hereafter, after the infiltration with pure epoxy resin for 3 h embedded in pure epoxy resin and cure in a 45 °C oven for 12 h, followed by a 72 °C oven for 24 h, the embedded blocks were sectioned to a thickness of 70 nm using a Leica UC-7 ultramicrotome. Then, the sections were collected on copper grids, dyed with lead citrate and photographed using a Japanese electron transmission microscope.

### Western blot

The proteins from islets and Min6 cells were lysed using lysis buffer containing protease and phosphatase inhibitors. Hereafter, the protein concentration was determined. The SDS-PAGE gel was prepared for separating proteins based on their molecular weight. Then, equal amounts of protein were loaded into the wells of the gel and subjected to electrophoresis. The proteins were transferred from the gel to a PVDF membrane, which was blocked to prevent non-specific binding using non-fat milk in TBST. Subsequently, the membrane was incubated with primary antibodies at the dilutions: LC3 (CST, 4108 s) P62(AF5384), P-ACC(AF3421), ACC(AF6421), P-mTOR (AF3308), mTOR (AF6308), P-AMPK (AF3423), AMPK (AF6423) all from Affinity at 1:1000; β-actin (Proteintech, 81115-1-RR) 1:10000; GAPDH (Proteintech, 10494-1-AP) 1:10000 overnight at 4 °C, followed by the further incubation with Anti-rabbit IgG, HRP-linked Antibody (CST, 7074) at 1:6000 dilution for 1 h at room temperature. The proteins were detected using chemiluminescent and the images of the blot were obtained using a chemiluminescence detection system. The bands were analyzed in the end using densitometry to quantify protein expression.

### Medicated serum preparation

Male Sprague–Dawley rats, 230–250 g, were divided into 2 groups: normal serum control and Cai’s herbal tea formula serum group (3.6 g/kg/d), with 6 in each group. The drug was orally administrated to rats. Before administration, the rats were fasted without water restriction. This procedure continues for 7 days. For the rats of normal serum control group, equal volume of saline was given. After the last dose, 5–10 mL of blood was harvested from decapitated rats in sterile tubes (without anticoagulant) and let stand at room temperature for 2 h, followed by centrifugation at 1500 r/min for 10 min and collection of supernatant. The serum was filtered through a 0.2 μm filter, aliquoted into 1 mL cryotubes, labeled, and stored at -80 ℃.

### Cell culture and grouping

Min6 cells (CL0461) were supplied by the Fenghui Biotech. High glucose Dulbecco’s Modified Eagle Medium (DMEM) (FI101-01, TRANSGEN BIOTECH) with 10% fetal bovine serum (FS301-02, TRANSGEN BIOTECH) and 1% penicillin–streptomycin solution (FG101-01, TRANSGEN BIOTECH) were mixed as needed. First, a high-glucose (HG) diabetes model was induced. Min6 cells of Control group were treated with 5.6 mmol/L glucose, while those of HG group received the intervention of 30 mmol/L glucose. The treatment lasted for 48 h. Then, Cai’s herbal tea formula serum was diluted to different concentrations (5%, 10%, 15%, 20%) in the culture medium, which was applied to pre-treat Min6 cells for 24 h. Meanwhile, some Min6 cells were treated with Metformin (5 mg/L) for 24 h [30]. All cells were collected afterwards. Then the cells were divided into the control, control + cai 1 (5% serum), control + cai 2 (10% serum), control + cai 3 (15% serum), control + cai 4 (20% serum) groups. Subsequently, the cells were divided into the control, HG (High Glucose), HG + cai L (10% medicated serum), HG + cai M (15% medicated serum), HG + cai H (20% medicated serum), HG + Metformin (5 mg/L) groups.

### Plasmid construction and grouping

Briefly, Min6 cells were seeded in a six-well culture plate and maintained in complete medium. Upon reaching 50–60% confluence, the cells were transfected with control siRNAs and siRNA targeting AMPK using Lipofectamine 2000 according to the manufacturer’s protocol. After glucose treatment, plasmids were transfected into Min6 cells for 24 h, followed by treatment with Cai’s herbal formula-containing serum concentrations for another 24 h, and metformin intervention for 24 h. Subsequently, cells were collected for relevant experiments. The cells were divided into the HG, HG + cai, HG + cai + si-NC(siRNA Negative Control), HG + cai + si-AMPK (siRNA AMP-Activated Protein Kinase) groups. The siRNA sequences are as follow: Protein Kinase AMP-Activated Catalytic Subunit Alpha 1(Prkaa1)-Mus-1165; GGCACACCCTGGATGAATTAA, si-NC(TTAATTCATCCAGGGTGTGCC).

### 3-(4,5-Dimethylthiazol-2-yl)-2,5-diphenyltetrazolium bromide (MTT) assay

Min6 cells in the logarithmic growth phase are counted, digested with 0.25% trypsin, and then seeded in a 96-well plate at a density of 5 × 10^3^ cells per well. Each cell group is set up with six replicate wells. After drug intervention, 20 μL of prepared MTT solution (5 mg/mL) is added to each well, followed by incubation at 37 °C for 4 h. After centrifugation of the 96-well plate at 1500 rpm/min for 10 min, the culture medium is removed, and 100 μL of DMSO is added to each well. The plate is gently shaken for 10–20 min until the purple-blue crystals are completely dissolved. The absorbance of each well is then measured at a wavelength of 490 nm using a microplate reader.

### Glucose stimulated INS secretion (GSIS) method

Min6 cells are seeded in a 6-well plate at a density of 5 × 10^5^ cells per well and cultured in a normal complete medium for 24 h. After cell adherence, the cells are treated according to their respective groups for 48 h. Cells are washed once with PBS, the supernatant is discarded and then incubated in glucose-free KRBB buffer for 30 min. Cells are washed once with PBS, the supernatant is discarded and then incubated in KRBB buffer containing 3 mmol/L and 30 mmol/L glucose for 1 h, respectively. The supernatant is collected. INS levels in each group are measured using an INS assay kit, strictly following the instructions provided. Basal INS Secretion (BIS) represents INS secretion stimulated by KRBB buffer containing 3 mmol/L glucose. GSIS represents INS secretion stimulated by KRBB buffer containing 30 mmol/L glucose. The INS Secretion Index (ISI) is calculated as GSIS/BIS.

### Data analysis

SPSS 20.0 software was used. For multiple groups, if the quantitative data were normally distributed and homogeneity of variance was confirmed, One-way ANOVA was used for analysis, with further pairwise comparisons between groups conducted using Tukey’s test. If data were normally distributed but variance was not homogeneous, Dunnett’s T3 test or independent sample t-test was applied. The significance level was set at α = 0.05. All data are presented as mean ± standard deviation (M ± SD), with *P* < 0.05 considered statistically significant.

## Results

### Therapeutic efficacy of Cai’s herbal tea in diabetic mice through induction of autophagy via the AMPK-mTOR pathway

**Fig. 1:**
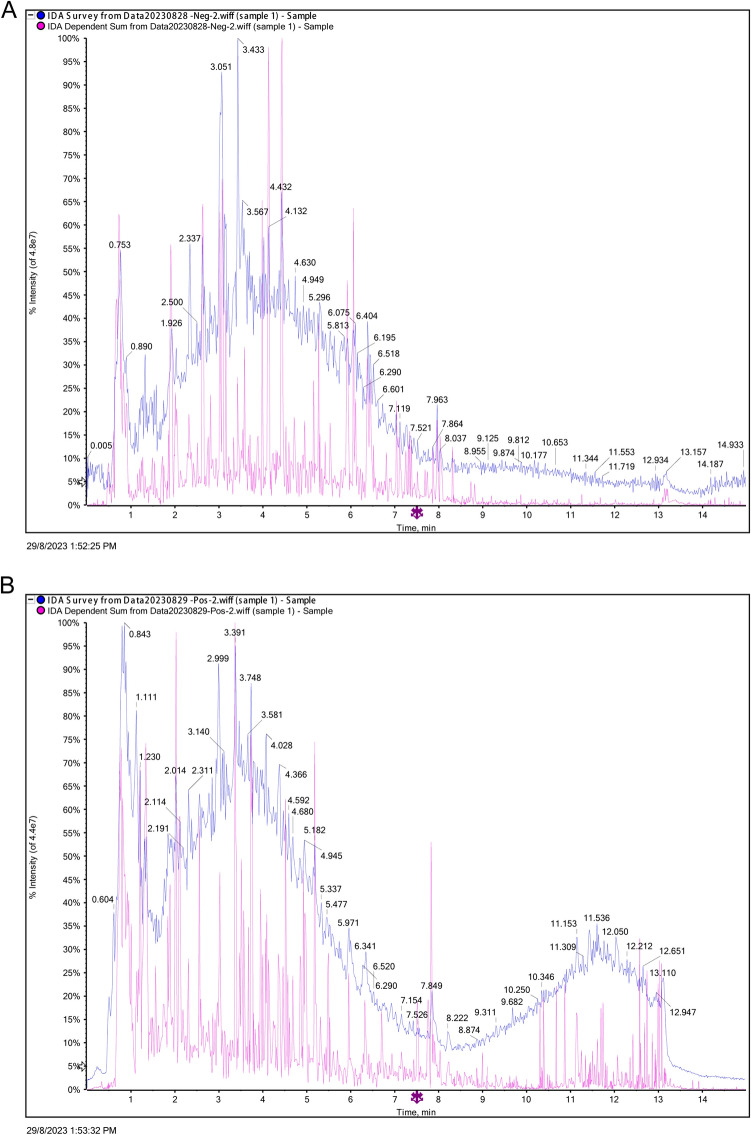
Component Analysis of Cai’s Herbal Tea Using UPLC-Q/TOF-MS. It presents the Total Ion Chromatogram obtained from Ultra-High Performance Liquid Chromatography-Quadrupole Time of Flight Mass Spectrometry (UPLC-Q/TOF-MS) analysis of Cai’s herbal tea formula. The chromatograms are depicted in two modes: **A** Positive Ion Mode and **B** Negative Ion Mode, showcasing the intricate ion profiles of the herbal constituents

In Fig. 1A, B, a comprehensive Analysis of Cai’s Herbal Tea Formula using UHPLC-Q/TOF-MS presents the total ion chromatogram obtained from UHPLC-Q/TOF-MS. The chromatograms are depicted in two modes: A) Positive Ion Mode and B) Negative Ion Mode, showcasing the intricate ion profiles of the herbal constituents. By comparing and screening with the compounds in the TCM MS/MS Library, which is a secondary database included in the SCIEX OS software, based on the criteria of primary accurate mass number (30%), isotopic distribution ratio (30%), and MS/MS spectra (40%), compounds with a matching score greater than 60% and a peak area exceeding 100 were identified. The results from this rigorous analysis revealed the presence of a multitude of compounds, reflecting the rich phytochemical composition of the herbal tea. Identified compounds included notable phytochemicals such as Calycosin, Calycosin-7-O-glucoside, Ononin, Atractylenolide I, Quercetin, Kaempferol, Rutin, Chlorogenic acid, and Methylophiopogonanone A (Supplementary Table 1).

**Fig. 2:**
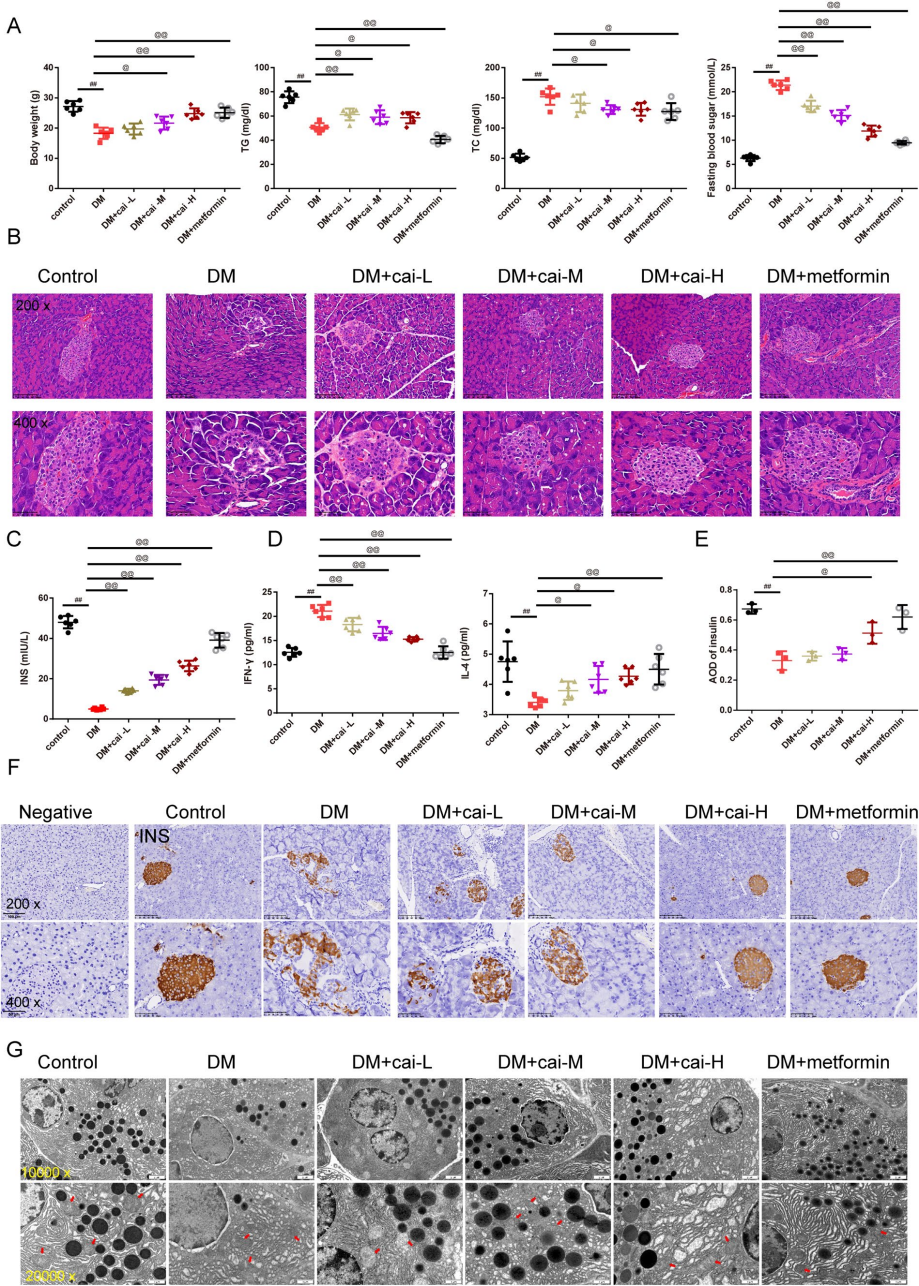
Therapeutic Efficacy of Different Doses of Cai’s Herbal Tea in Diabetic Mellitus (DM) Mice. **A** Key physiological parameters including body weight, triglycerides (TG), total cholesterol (TC), and fasting blood sugar levels of mice were quantitatively measured in a sample size of six (*n* = 6). **B** Hematoxylin and Eosin (HE) staining provided detailed insights into the pathological changes in the pancreatic islets of mice, with images captured at ×200 and ×400 magnifications (scale bars: 100 μm and 50 μm, respectively). **C**, **D** Enzyme-Linked Immunosorbent Assay (ELISA) kits were employed to determine the levels of insulin (INS), interferon-gamma (INF-γ, and interleukin-4 (IL-4) in the pancreatic islets of mice, with a sample size of six (*n* = 6). **E**, **F** Immunohistochemistry (IHC) staining highlighted the positive expression INS in the pancreatic islets of mice, visualized at ×200 and ×400 magnifications (scale bars: 100 μm and 50 μm, respectively), with a sample size of three (*n* = 3).G: Transmission Electron Microscopy (TEM) revealed subtle ultrastructural changes in the pancreatic islets of mice, with a focus on mitochondria (indicated by red arrows), captured at ×10.0 k and ×20.0 k magnifications (scale bars: 2 μm and 1 μm, respectively). Statistical significance is denoted as ^##^*P* < 0.01 versus the control group, and ^@^*P* < 0.05, ^@@^*P* < 0.01 versus the DM group

**Fig. 3:**
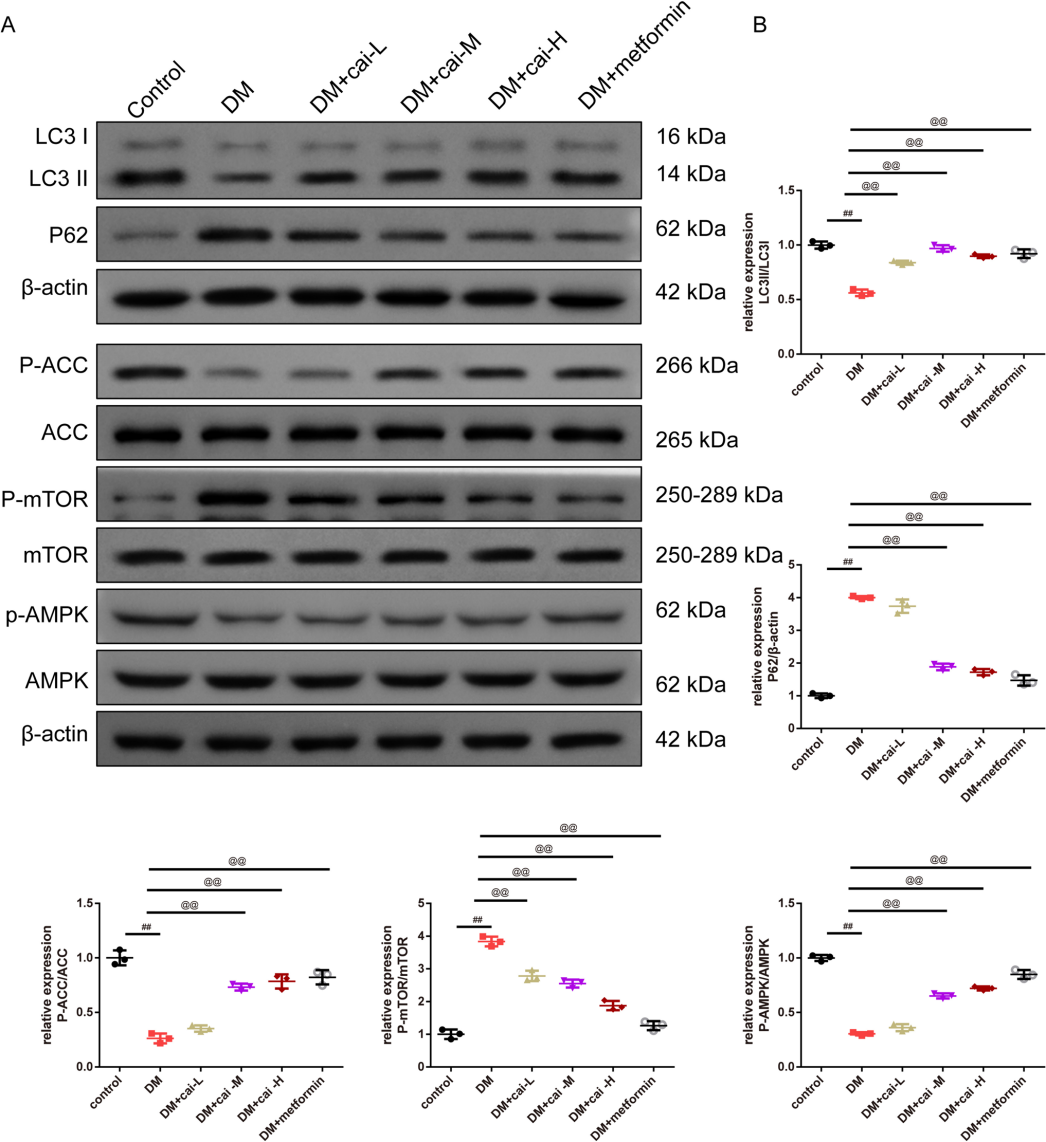
Therapeutic Efficacy of Cai’s Herbal Tea in DM Through Induction of Autophagy via the AMPK-mTOR Pathway. **A**, **B** Western blot analysis was conducted to observe the expression levels of LC3 I, LC3 II, P62, phosphorylated Acetyl-CoA Carboxylase (P-ACC), ACC, phosphorylated mammalian Target of Rapamycin (P-mTOR), mTOR, phosphorylated AMP-activated Protein Kinase (P-AMPK), and AMPK. The sample size for this analysis was three (*n* = 3), with statistical significance indicated as ^##^*P* < 0.01 versus the control group and ^@@^*P* < 0.01 versus the DM group

As shown in Fig. 2A, key physiological parameters including body weight, TG, TC, and fasting blood sugar levels were quantitatively measured. The results demonstrated that DM mice had higher body weight, TC, and fasting blood sugar whereas lower TG than control mice (*P* < 0.01). Different doses of Cai’s Herbal Tea acted conversely in a dose-dependent way(*P* < 0.05). The addition of metformin exhibited the best therapeutic effect (Fig. 2A). In Fig. 2B, HE staining provided detailed insights into the pathological changes in the pancreatic islets of mice. The islet tissue of control mice appears intact with normal islet area and cell count. In contrast, the islet tissue of DM and DM + cai-L mice show extensive cell necrosis and dissolution, with a noticeable reduction in islet area and number, indicating severe damage. The islet tissue in DM + cai-M, DM + cai-H, and DM + metformin mice exhibit improvement in necrosis and dissolution, with an increase in islet area and number. In Fig. 2C, D, ELISA kits employed to determine the levels of INS, INF-γ, and IL-4 in the pancreatic islets showed that DM mice had higher INF-γ whereas lower INS, IL-4 than control mice (*P* < 0.01). Different doses of Cai’s Herbal Tea acted conversely in a dose-dependent way(*P* < 0.05). The addition of metformin exhibited the best therapeutic effect (Fig. 2C, D). In Fig. 2E, F, IHC staining showed that compared to the control, the AOD value of INS in the islet tissue of DM mice significantly decreased, indicating a significant reduction in expression (*P* < 0.01). Compared to DM, the AOD value of INS in the islet tissue of DM + cai-H and DM + metformin mice significantly increased, indicating a significant increase in expression (*P* < 0.05 or *P* < 0.01). In Fig. 2G, TEM revealed subtle ultrastructural changes in the pancreatic islets, with a focus on mitochondria (indicated by red arrows). The mitochondria in the islet β-cells of control mice appear normal in morphology, with clear and intact cristae, and numerous secretory granules are visible. In DM mice, islet β-cells exhibit mitochondrial swelling, blurred cristae, and fewer secretory granules. Compared to DM, the mitochondrial damage in islet β-cells of DM + cai-L, DM + cai-M, DM + cai-H, and DM + metformin mice is reduced to varying degrees, with clearer cristae and an increased number of secretory granules. In Fig. 3A, B, Western blot analysis revealed that Cai’s Herbal Tea induced higher expression of the ratio of LC3 II/LC3 I, P-ACC/ACC, P-AMPK/AMPK whereas the lower expression of the ratio of P-mTOR/mTOR, P62 of the pancreas islet in DM mice than control mice in a dose-dependent way(*P* < 0.01). The interfere of the metformin on the DM mice exerted better effect.

### Therapeutic efficacy of Cai’s herbal tea in high-glucose treated Min6 cells through promoting autophagy via the AMPK-mTOR pathway

**Fig. 4:**
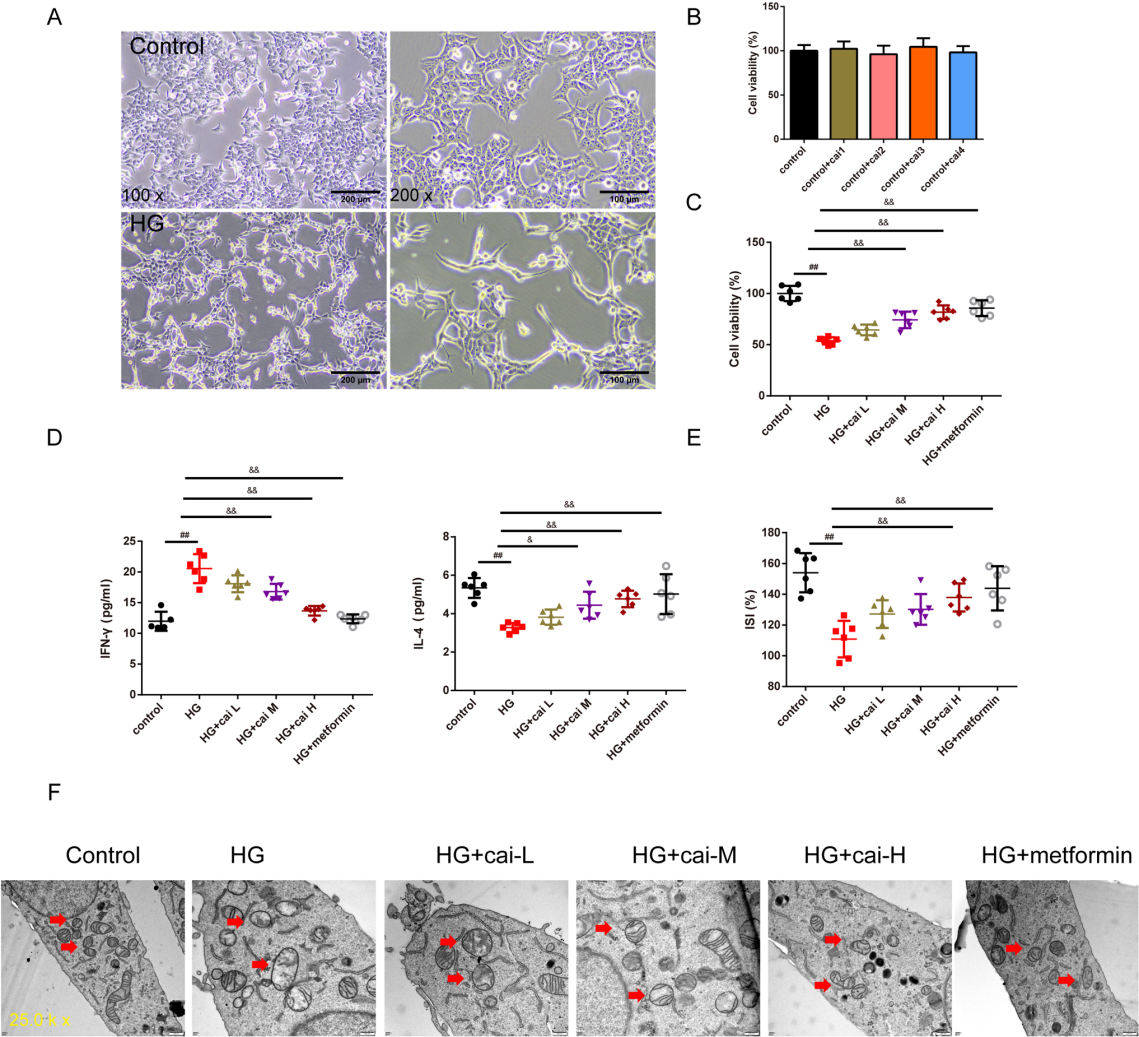
Efficacy of Different Doses of Cai’s Herbal Tea in Promoting Autophagy in High-Glucose Treated Min6 Cells. **A** Bright field microscopy was used to observe the morphological changes in Min6 cells at ×100 and ×200 magnifications (scale bars: 200 μm and 100 μm, respectively). **B**, **C** Cell viability was rigorously evaluated in Min6 cells treated with four different concentrations of Cai’s herbal formula (5%, 10%, 15%, and 20% medicated serum). Additionally, cells were subjected to high-glucose (HG) stress at 30 mmol/L, with three levels of medicated serum (10%, 15%, and 20% labeled as Low, Medium, and High concentrations, respectively) and compared with a metformin treatment group (5 mg/L). The MTT assay, a reliable method for assessing cell metabolic activity, was conducted over a 24-h period, with a sample size of six (*n* = 6). **D** ELISA kits quantified the levels of INF-γ, and IL-4 in the pancreatic islets, with a sample size of six (*n* = 6). **E** The Glucose-Stimulated INS Secretion (GSIS) assay was employed to evaluate the INS release from Min6 cells following high-glucose intervention (The INS Secretion Index (ISI) is calculated as the ratio of GSIS to Basal INS Secretion (BIS)), with a sample size of six (*n* = 6). **F** TEM provided detailed visualization of the minor changes in Min6 cells, focusing on mitochondria (indicated by red arrows), at magnifications of ×25.0 k (scale bars: 500 nm). Statistical significance is denoted as ^##^*P* < 0.01 versus the control group, ^&^*P* < 0.05, and ^&&^*P* < 0.01 versus the HG group

**Fig. 5:**
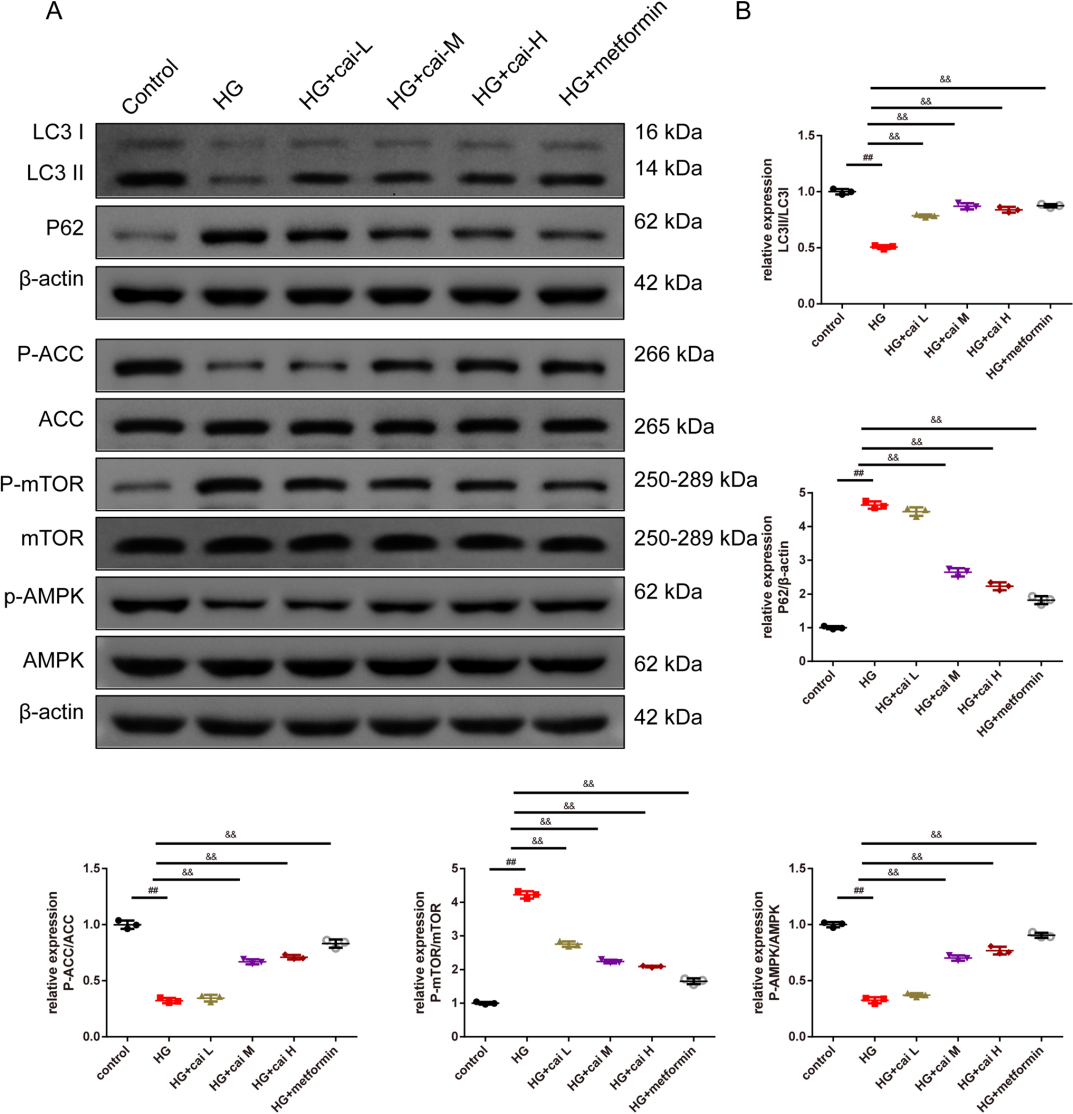
Therapeutic Efficacy of Cai’s Herbal Tea in High-Glucose Treated Min6 Cells Through Promoting Autophagy via the AMPK-mTOR Pathway. **A**, **B** Western blot analysis was used to observe the levels of LC3 I, LC3 II, P62, P-ACC, ACC, P-mTOR, mTOR, P-AMPK, and AMPK. The sample size for this analysis was three (*n* = 3), with statistical significance indicated as ^##^*P* < 0.01 versus the control group and ^&&^*P* < 0.01 versus the HG group

**Fig. 6:**
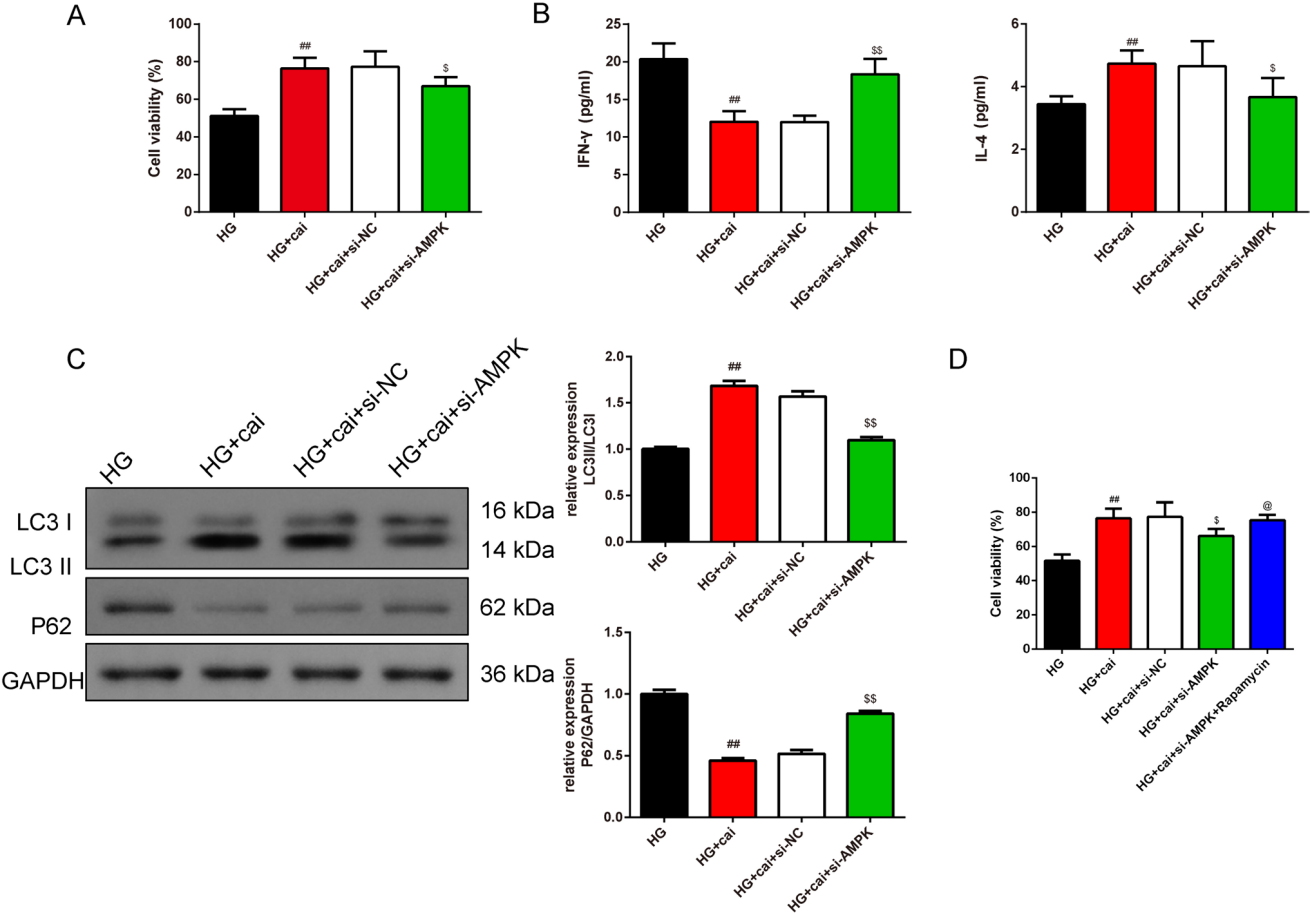
Therapeutic Efficacy of Cai’s Herbal Tea in High-Glucose Treated Min6 Cells Through Promoting Autophagy via the AMPK-mTOR Pathway. **A** MTT assay evaluated the cell viability of Min6 cells treated with Cai’s formula and metformin over 24 h, with a sample size of six (*n* = 6). **B** ELISA kits assessed the levels of INF-γ, and IL-4 in Min6 cells, with a sample size of six (*n* = 6). **C** Western blot analysis observed the protein expression of LC3 I, LC3 II, P62 in Min6 cells, with a sample size of three (*n* = 3). **D** MTT assay evaluated the cell viability of Min6 cells treated with rapamycin over 24 h, with a sample size of six (*n* = 6). Statistical significance is indicated as ^##^*P* < 0.01 versus the HG group, ^$^*P* < 0.05, and ^$$^*P* < 0.01 versus the HG + cai + si-NC group, ^@^P < 0.05, versus the HG + cai + si-AMPK group

In Fig. 4A, the bright field microscopy was used to observe the morphological changes in Min6 cells. In the control group, the cells exhibited an epithelial-like morphology, adhering to the wall and predominantly assuming a polygonal shape. In the HG group, the cells underwent morphological changes, becoming smaller and more rounded, with some elongating. There was an increase in intercellular spacing and a reduction in the number of cells adhering to the wall. In Fig. 4B, cell viability was rigorously evaluated in Min6 cells showed that there was no significant difference in four Cai’s herbal formula (5%, 10%, 15%, and 20% medicated serum) groups. Additionally, in Fig. 4C, the MTT assay results showcased that HG results in the lower cell viability. Different doses of Cai’s Herbal Tea (10%(L), 15%(M), and 20%(H) medicated serum) enhanced cell viability of the Min6 cells in a dose-dependent way (*P* < 0.05). The addition of metformin exhibited the best therapeutic effect (*P* < 0.01). In Fig. 4D, E, ELISA kits and GSIS assay quantified the levels of IFN-γ and IL-4 INS release from Min6 cells. The results showed that HG group had higher INF-γ whereas lower INS, IL-4 than control cells (*P* < 0.01). Different doses of Cai acted conversely in a dose-dependent way(*P* < 0.05). The addition of metformin exhibited the best therapeutic effect (*P* < 0.01). In Fig. 4F, TEM results show that the mitochondrial structure in Min6 cells of the control group is essentially normal. Compared to the control group, the mitochondria in the HG group’s Min6 cells are noticeably swollen, with a significant reduction in cristae. In the HG + cai L group, the reduction in cristae is alleviated; in the HG + cai M, HG + cai H, and HG + metformin groups, mitochondrial swelling is significantly reduced, and the reduction in cristae is significantly alleviated. In Fig. 5A, B, Western blot analysis confirmed that Cai’s Herbal Tea induced higher expression of the ratio of LC3II/LC3I, P-ACC/ACC, P-AMPK/AMPK whereas the lower expression of the ratio of P-mTOR/mTOR, P62 in Min6 cells than control group in a dose-dependent way (*P* < 0.01). The interfere of the metformin on the HG group exerted better effect (*P* < 0.01). In Fig. 6A–C, MTT assay, ELISA kits, and Western blot reconfirmed that HG + cai group had higher cell viability, expression of INF-γ, IL-4 and the ratio LC3II/ LC3I whereas the lower expression of P62 than HG group (*P* < 0.01). Additionally, with the addition of the si-AMPK, the above effects were conversed compared with HG + cai + si-NC group (*P* < 0.05 or *P* < 0.01). In Fig. 6D, compared to the HG group, β-cell activity was significantly increased in the HG + cai group (*P* < 0.01). No significant difference in β-cell activity was observed between the HG + cai group and the HG + cai + si-NC group (*P* > 0.05). Compared to the HG + cai + si-NC group, β-cell activity was significantly decreased in the HG + cai + si-AMPK group (*P* < 0.05). Furthermore, compared to the HG + cai + si-AMPK group, β-cell activity was significantly reduced in the HG + cai + si-AMPK + Rapamycin group of mice (*P* < 0.05).

## Discussion

The study’s findings significantly enhance our understanding of Cai’s Herbal Tea’s therapeutic efficacy in managing T1DM, particularly through the induction of mitochondrial autophagy via the AMPK-mTOR pathway. The comprehensive analysis of Cai’s Herbal Tea using UHPLC-Q/TOF-MS identified key compounds that align with those in the TCM MS/MS Library. The results from this rigorous analysis revealed the presence of a multitude of compounds, reflecting the rich phytochemical composition of the herbal tea. Identified compounds included notable phytochemicals such as Calycosin, Calycosin-7-*O*-glucoside, Ononin, Atractylenolide I, Quercetin, Kaempferol, Rutin, Chlorogenic acid, and Methylophiopogonanone A. These compounds are known for their various therapeutic properties, ranging from anti-inflammatory and antioxidant activities to potential benefits in modulating immune responses, metabolic processes and diabetics [31–33]. Furthermore, the analysis also highlighted the presence of other significant compounds like Hyperin, Luteolin, Berberine, and Matrine, each contributing to the holistic therapeutic potential of Cai’s Herbal Tea Formula. The diversity of these compounds underscores the complexity of TCM formulations and their multifaceted approach to diabetics [34, 35].

The dose-dependent improvements in physiological parameters such as body weight, TG, TC, fasting blood sugar levels and inflammatory response highlighting the tea’s potential in managing critical aspects of T1DM are consistent with previous studies [36, 37]. In addition, a randomized controlled trial explored the combined effects of exercise and TCM herbal tea in patients with DM, suggesting potential benefits in glycemic control [38]. Notably, *Atractylodes* (one of the components of Cai’s Herbal Tea) has been reported to inhibit inflammation in diabetic rats, further underscoring the tea’s anti-inflammatory properties [39].

The histological improvements observed in the pancreatic islets of DM mice treated with different doses of Cai’s Herbal Tea are noteworthy. The reversal of cell necrosis and dissolution, and the restoration of islet area and cell count, suggest a significant protective effect on pancreatic beta cells. Apart from that, a study reported that *Astragalus polysaccharides* (one of the components of Cai’s Herbal Tea)-administered non-obese diabetic (NOD) mice had a lower incidence rate of T1DM, lower serum C-peptide level, better histologic findings of pancreatic islets [40]. This protection is crucial, considering the central role of beta cells in diabetes pathophysiology [41]. The observed morphological changes in Min6 cells under HG conditions are consistent with the cellular stress and apoptosis reported in diabetic conditions [42]. In the Min6 cell model, Cai’s Herbal Tea demonstrated a protective effect against HG-induced cytotoxicity. The observed morphological changes and the enhancement of cell viability in a dose-dependent manner underscore the tea’s potential in mitigating hyperglycemia-induced cellular damage. This is further supported by the ELISA and GSIS assay results, which showed improved INS and cytokine profiles in the treated cells.

Our TEM results showing mitochondrial swelling and blurred cristae in diabetic conditions align with studies indicating mitochondrial dysfunction in pancreas islet of the diabetic mice. Studies on mitochondrial-derived peptides in diabetes elucidates the role of mitochondrial dysfunction in worsening INS resistance and promoting apoptosis [43, 44]. The improvement in mitochondrial morphology with Cai’s Herbal Tea suggests its potential in mitigating these dysfunctions, possibly through enhancing mitochondrial biogenesis. A study reported that the diabetic mice exhibited higher levels of autophagy compared to wild-type mice. In addition, mitochondrial function was improved in diabetic mouse after polydatin treatment [45]. The Western blot analysis suggest that Cai’s Herbal Tea may exert its effects by enhancing autophagic activity, thereby improving cellular function and survival in diabetic conditions. The enhancement of autophagic activity as evidenced by the increase in LC3 II/LC3 I ratio and decrease in P62 expression, is a critical finding. Consistently, Hu et.al, highlighted the importance of autophagy in mitigating oxidative stress and improving mitochondrial function in diabetic conditions [46]. Chen proved that *Astragalus polysaccharide* promotes autophagy and alleviates diabetic nephropathy [47].

The role of the AMPK-mTOR pathway in diabetes management has been highlighted in several studies. In our study, the protective effect of Cai in the HG-induced Min6 cells was conversed by addition of si-AMPK. Research on the Yangxinkang tablet, a Chinese herbal compound, shows its protective effects against cardiac dysfunction through the inhibition of AMPK/mTOR-mediated autophagy [48]. Furthermore, the study emphasizes the modulation of the AMPK pathway by TCM to improve metabolism, autophagy, and reduce oxidative stress [49].

Cai’s Herbal Tea demonstrates significant therapeutic efficacy in T1DM management, primarily through the induction of autophagy via the AMPK-mTOR pathway. These findings are supported by recent research, suggesting that traditional herbal medicines can offer valuable and effective treatment options for managing complex diseases like T1DM. However, while these results are promising, several limitations must be addressed through further research. Specifically, there is a need for comprehensive clinical trials to verify the long-term efficacy and safety of such herbal treatments. These studies should aim to establish standardized dosing regimens, identify potential side effects, and understand the interactions of herbal compounds with conventional T1DM medications. Additionally, the variability in herbal medicine composition due to differing growth conditions, harvest times, and preparation methods necessitates the development of quality control standards to ensure consistency and reliability of treatment outcomes. The biological mechanisms underlying the therapeutic effects observed also warrant deeper investigation to fully appreciate how these herbal compounds interact at the cellular and molecular levels. Only through such detailed and rigorous investigation can the full potential of traditional herbal medicines like Cai’s Herbal Tea be realized and safely integrated into the broader spectrum of T1DM management strategies.

## Conclusion

In summary, the findings of the study indicate that at higher doses, Cai’s Herbal Tea significantly improved body weight, pancreatic islet pathology, and insulin secretion, while reducing TG, fasting blood sugar levels, and INF-γ in T1DM mice. In Min6 cells, the herbal tea mitigated high glucose-induced damage and pro-inflammatory responses, enhancing cell viability and insulin secretion capabilities. Importantly, the study unveiled the molecular mechanisms by which Cai’s Herbal Tea promotes autophagy through the AMPK-mTOR pathway. This was evidenced by reduced mitochondrial swelling, improved cristae structure in the treated groups, an increase in the LC3II/LC3I and P-AMPK/AMPK ratios, and a decrease in P-mTOR/mTOR and P62 expressions in pancreatic islet β-cells. Furthermore, the critical role of AMPK in this process was confirmed through si-AMPK interference. Cai’s Herbal Tea demonstrates significant therapeutic efficacy in T1DM mice by enhancing mitochondrial health and inducing autophagy in pancreatic islet β-cells through the AMPK-mTOR pathway. These findings underscore its potential as a therapeutic approach for the management of T1DM, paving the way for further clinical research and development.

## Supplementary Information

Below is the link to the electronic supplementary material.

Supplementary file1 (XLSX 26 KB)

## Data Availability

The datasets generated during and/or analyzed during the current study are available from the corresponding author upon reasonable request.

## Abbreviations

T1DM: Type 1 diabetes mellitus
INS: Insulin
IL-1β: Interleukin-1β
MAP1LC3: Microtubule-associated protein 1 light chain 3
AMPK: AMP-activated protein kinase
UPLC-Q/TOF-MS: Ultra performance liquid chromatography-quadrupole/time of flight-mass spectrometry
IDA: Information dependent acquisition
TG: Total triglycerides
TC: Total cholesterol
ELISA: Enzyme-linked immunosorbent assay
INF-γ: Interferon gamma
IL-4: Interleukin 4
HE: Hematoxylin and eosin
IHC: Immunohistochemistry
TEM: Transmission electron microscopy
DMEM: Dulbecco’s modified eagle medium
HG: High-glucose
si-NC: SiRNA negative control
si-AMPK: SiRNA AMP-activated protein kinase
MTT: 3-(4,5-Dimethylthiazol-2-yl)-2,5-diphenyltetrazolium bromide
GSIS: Glucose stimulated INS secretion
BIS: Basal INS secretion
ISI: INS secretion index
NOD: Non-obese diabetic
